# Case report of acute hepatorenal failure induced by third-line treatment with tislelizumab in a patient with cholangiocarcinoma: was influenza virus the culprit?

**DOI:** 10.3389/fimmu.2025.1631953

**Published:** 2025-12-11

**Authors:** Xuebing Zhang, Xia Zhang, Hang Yin, Weiguo Zhang, Bin Zhang

**Affiliations:** 1 Department of Oncology, The First Affiliated Hospital of Dalian Medical University, Dalian, Liaoning, China; 2 Department of Oncology, Dalian Fifth People’s Hospital, Dalian, Liaoning, China

**Keywords:** immune-related adverse events, tislelizumab, H1N1 influenza A virus (IAV), immune storm, intrahepatic cholangiocarcinoma (ICC)

## Abstract

A 72-year-old male diagnosed with cholangiocarcinoma was initiated on third-line therapy comprising Tislelizumab and Anlotinib. Within four days of treatment, he developed fulminant hepatic injury concurrent with acute kidney injury. Despite aggressive management with high-dose glucocorticoids, hepatoprotective agents, comprehensive supportive care, and subsequent anti-infective therapy, his clinical status declined rapidly. In view of the grave prognosis and the family’s decision to decline intensive interventions such as plasma exchange, the patient ultimately succumbed to multiorgan failure. This case highlights a potential synergistic interaction between concomitant infection (e.g.,influenza virus) and immune checkpoint inhibitor(ICI) therapy, possibly mediated through enhanced antigenic stimulation and loss of immunoregulatory control, culminating in exaggerated immune activation. This mechanism may have profoundly amplified ICI-related toxicity, leading to fatal multiorgan irAEs. Regarding the issue of immune storms, it is challenging for clinical practice to provide favorable outcomes for patients, and we need to remain highly vigilant.

## Introduction

Intrahepatic cholangiocarcinoma (ICC) is the second most common primary liver tumor,with a global incidence of 0.3-6/100,000 and a higher incidence in China (>6/100,000). The global mortality rate is 1-6/100,000, while in China,it is >4/100,000. Most patients (70%) are diagnosed at an advanced stage, with a 5-year survival rate of 7-20%. Approximately 20%-30% of patients are eligible for surgery, which is the only potentially curative treatment; adjuvant capecitabine after surgery has a median survival of 53 months. For the 70%-80% of patients with locally unresectable or metastatic disease,systemic therapy may delay progression,but survival is limited to approximately 1 year (1). For the past decade, doublet chemotherapy with gemcitabine and cisplatin has been considered the most effective first-line regimen, but the results of ICI therapy may shift this paradigm for the first time (2). For patients who have not received ICI therapy initially, second-line treatment with the addition of immunotherapy may improve patient prognosis. However, the adverse reactions of ICI are still unpredictable, and events leading to death have occurred in both clinical studies and the real world, although the proportion is extremely low, it is still a problem that we need to pay close attention to. Cytokine storm is the main cause of death, but it is sometimes difficult to detect clinically. This article reports a case of a patient with influenza virus infection during ICI therapy, who developed severe liver damage and eventually died of unknown causes, hoping to raise clinical vigilance.

It is worth noting that the bottleneck of the therapeutic effect of cholangiocarcinoma is partly due to its complex tumor microenvironment and the lack of specific targeting methods. In order to overcome this limitation, A study revealed that that single-cell multi-omics is helpful to discover new therapeutic targets for cholangiocarcinoma (3). Rishabha et al. have reviewed how to exploit the unique electrical, optical and magnetic properties of cancer cells to develop new therapies. By intervening these physical parameters, strategies such as magnetic field-assisted therapy play an important role in improving the effectiveness and safety of cancer diagnosis and treatment (4). At the same time, the rise of nanotechnology has brought another powerful impetus to achieve precision medicine. As multifunctional therapeutic and diagnostic platforms, novel photoactivated nanomaterials have shown great potential in the field of cancer. Although it is not yet mature from laboratory research to large-scale clinical application, such cutting-edge technologies undoubtedly lay an innovative foundation for overcoming the dilemma of solid tumor treatment, including cholangiocarcinoma (5).

## Case data

In December 2022,a 72-year-old male presented to the Second Affiliated Hospital of Dalian Medical University with abdominal distension and scleral icterus. He had no history of smoking and drinking. He did not have a medical history of hypertension, diabetes, kidney disease, or hepatitis. and no family members had a tumor history.

Imaging revealed thickening of the mid-common bile duct wall, raising suspicion of malignancy. On February 3,2023, the patient underwent radical resection of the hilar bile duct carcinoma, retroperitoneal lymph node dissection, and complex adhesion release under general anesthesia. Pathology confirmed moderately to poorly differentiated extrahepatic cholangiocarcinoma, T2N0Mx.

The immunohistochemical examination indicated pMMR, CK7(+), Muc-1(+), CK20(+), CDx2(-), Muc-5(+)Ki67(35%+), PMS2(+), HER-2(-). Genetic testing revealed microsatellite stability (MSS),TMB 0.53 Muts/Mb,PD-L1(-), absence of ERBB-2 amplification, and strong VEGFR expression. Postoperative radiotherapy was initiated in March 2023,concurrently with capecitabine 1000mg po bid. The patient received four cycles of single-agent tegafur in May 2023,followed by three cycles of gemcitabine plus cisplatin due to disease progression in September. The patient went to our hospital on March 28,2024,due to further disease progression. Based on previous findings, the diagnosis was cholangiocarcinoma with peritoneal lymph node metastasis. A third-line treatment regimen of tislelizumab combined with anlotinib was planned. The patient’s ECOG score was 2 before treatment, and the patient’s laboratory results on admission are summarized in **Table 1**.

**Table 1:** The patient's laboratory results on admission.

Laboratory test	Patient’s value	Reference range	Unit
WBC(White blood cell)	3.17	3.5-9.5	10^9/L
LYMPH(Lymphocyte percentage)	16.1	20-50	%
MONO(Monocyte percentage)	12.60	3-10	%
RBC(Red blood cell)	3.06	4.3-5.8	10^12/L
HB(Hemoglobin)	101	130-175	G/L
PLT(Blood platelet)	94	125-350	10^9/L
ALT(Alanine Aminotransferase)	30	9-50	U/L
AST(Aspartate Aminotransferase)	55	15-46	U/L
T-BIL(Total Bilirubin)	13.7	3-22	μmol/L
CK-MB(Creatine Kinase)	0.45	<5	μg/L
MYO(Myoglobin)	47.27	<110	ng/ml
Hs-TnI(High-sensitivity troponin I)	0.003	0-0.057	μg/L

The patient developed a fever on the night of March 29,the day of toripalimab initiation, with a peak temperature of 39.5°C,accompanied by mild catarrhal symptoms. Upper respiratory tract infection was suspected,and symptomatic treatment was administered, resulting in a decrease in temperature. However,the fever recurred. On the fourth day of medication (April 1) the patient experienced another high fever. A comprehensive etiologic workup was performed on April 2nd during the acute phase of his clinical deterioration.Respiratory pathogen testing was performed,and the COVID-19 antigen test was negative.

Laboratory results from a 6:00 AM blood draw revealed a neutrophil count of 8.09×10*9,a platelet count of 28×10*9,hemoglobin of 147g/L, and WBC:4.63×10*12. The neutrophil percentage was 78%, and the lymphocyte percentage was 14.4%.

Liver function:ALT:353U/L, AST:1095U/L, T-BIL:33.3μmol/L,Creatinine(CRE):43umol/L(normal:58-110umol/L), procalcitonin (PCT): 6.68 ng/ml(normal:0-0.5ng/ml), C-reactive protein(CRP): 104.68 mg/L(normal:0-6mg/L), which was suggestive of immune checkpoint inhibitor-related hepatitis. Intravenous infusion of methylprednisolone (160mg per day) and glutathione (2.4mg per day) and diphenhydramine (20mg once) as well as oral bicyclol (25mg once) were immediately administered.

Repeat liver function tests at 9:00 AM showed ALT: 467 U/L,AST: 1501 U/L,ALP: 231 U/L,T-BIL: 36 μmol/L,and conjugated bilirubin 8.3 μmol/L; Albumin: 24.5 g/L,indicating rapidly deteriorating liver function.CK-MB: 12.84 μg/L,hs-tnI: 0.165 μg/L,MYO: 1238.55 ng/ml,which was suggestive of myositis,with a potential for myocardial injury,hypoalbuminemia,and thrombocytopenia. Treatment included liver protective,pulse hormone therapy (methylprednisolone 340mg per day), anti allergic (diphenhydramine 20mg once),platelet transfusion,and albumin supplementation.

Physical examination showed that the jaundice of the patient’s skin worsened rapidly in a short period of time, with poor appetite, extreme fatigue, irritability, abdominal distension, and no nausea or vomiting. The patient could not cough up phlegm and was given phlegm and cough relieving treatment. The patient had dysuria, and diuretic therapy was intensified. The family was informed,and liver replacement therapy was recommended,but they opted for pharmacological treatment due to financial constraints.

On the April 2th the patient’s condition worsened,with 24-hour urine output <500 ml. Repeat tests revealed CRP: 83.82 mg/L,WBC:12.93 10^9/L, LYMPH: 18.30%, MONO:17.90%, CK-MB:34.19 μg/L, hs-TnI:0.431 μg/L, MYO:1080.17 ng/ml, ALT: 743 U/L, AST: 2752 U/L, ALP: 225 U/L, T-BIL: 381 μmol/L, CRE:168 μmol/L. The patient presented with liver failure,renal insufficiency, and oliguria, suggestive of Multiple Organ Dysfunction Syndrome (MODS). Plasma exchange was again recommended but declined by the family. Concurrently, pathogen reports indicated positive IgM antibodies for influenza A virus, influenza B virus, and Legionella pneumophila. The patient was treated with antiviral therapy and anti-inflammatory therapy with marbasalovir following consultation with the intensive care unit.

**Figure 1:**
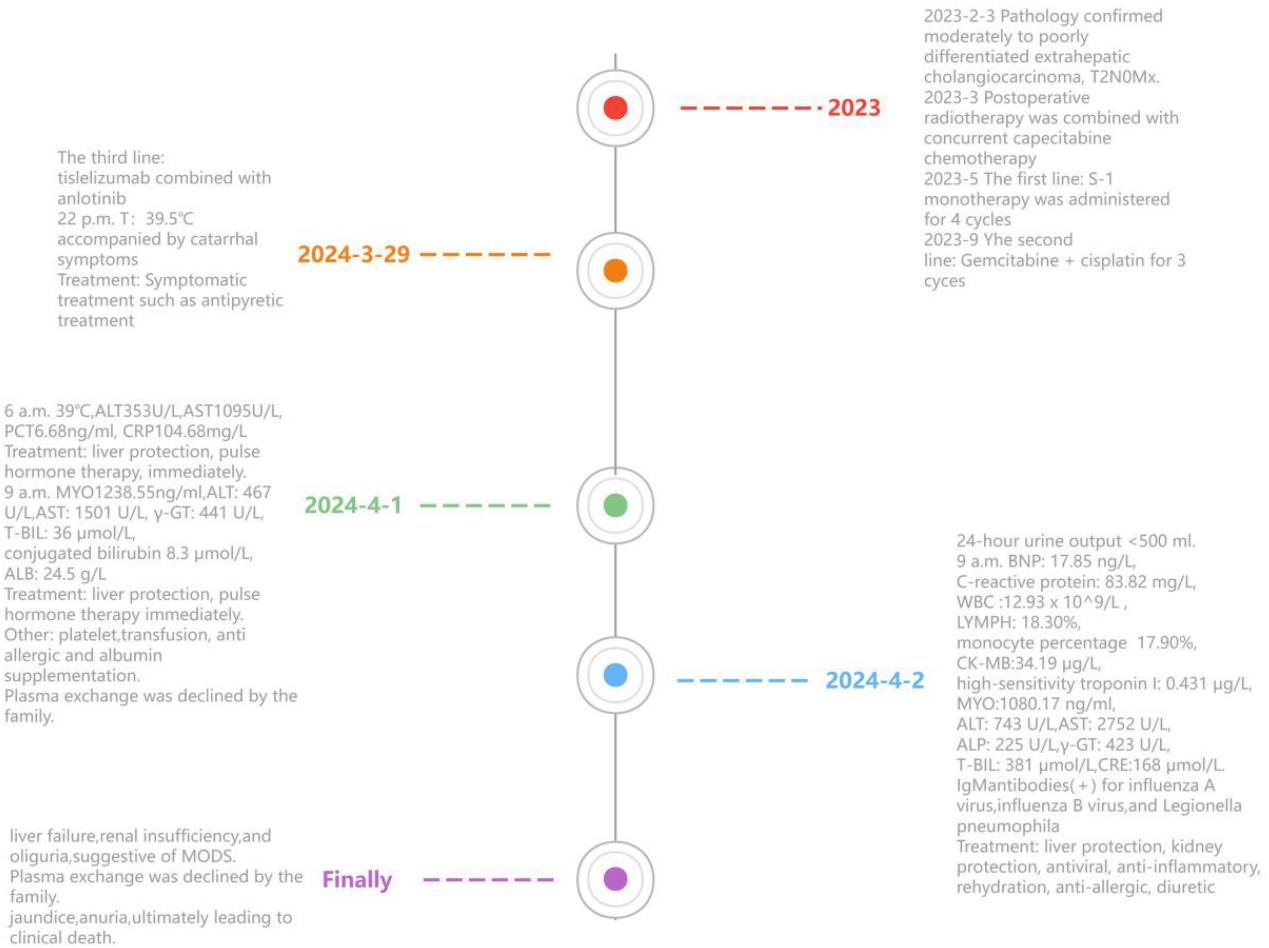
The timeline of the patient’s symptom, diagnosis and treatments.

Intravenous infusion of Shenkang injection (100ml per day), fluid replacement, intravenous injection human immunoglobulin (10mg iv), compound amino acid injection (500ml per day), and levofloxacin (500mg per day), pheresis leucopenic platelets(iv), oral mabasalovir (40mg once), Anuria persisted despite repeated use of diuretics. However, the patient’s condition rapidly progressed, with the development of jaundice and anuria, ultimately leading to clinical death on the April 3. The timeline of symptoms, diagnosis, and treatment was summarized in **Figure 1**.

## Discussion

Analysis of the cause of death indicated that the underlying fatal event was fulminant,lethal immune-related adverse events (irAEs) induced by combined ICI therapy and antiangiogenic therapy. Subsequent serologic testing revealed positive IgM antibodies against influenza virus and Legionella species,which suggesting recent coinfection as potential contributory factors. Anlotinib, a multitargeted tyrosine kinase inhibitor (TKI), is associated with hepatotoxic effects—including elevated transaminases and hyperbilirubinemia—as described in its instruction. The hepatotoxicity profile of anlotinib monotherapy is generally characterized by low-grade, asymptomatic elevations in transaminases, triglycerides, total cholesterol, and bilirubin (6). Mechanistically, anlotinib may alter the tumor immune microenvironment by inhibiting vascular endothelial growth factor receptor (VEGFR) signaling or downregulating PD-L1 expression on vascular endothelial cells (7). When combined with immune checkpoint inhibitors (ICIs), these effects may synergistically enhance systemic T-cell activation, thereby amplifying on-target, off-tumor toxicity in normal tissues such as the liver. However, the current clinical trials of ICI + TKI combination therapy have not reported a higher incidence of immune-related adverse events (irae), especially hepatitis, than that of anlotinib monotherapy (8). According to the known clinical data, the side effects of immunotherapy combined with anti-angiogenesis therapy can not be prevented, but are basically controllable. Thus, while anlotinib monotherapy is unlikely to fully account for the fulminant hepatic failure observed in this case, it may have acted as a “toxicity amplifier,”potentiating the immune-related toxicity of tislelizumab. An acute influenza infection may have served as a third insult, further exacerbating this dysregulated immune response. The Naranjo Adverse Drug Reactions Probability Scale was used for monitoring adverse drug reactions (9), the patient’s score of 6 points showed that the causal relationship between tislelizumab and irAE was ‘probable’ (**Table 2**). We speculate that acute influenza virus infection may have acted as a powerful catalyst in this process.

**Table 2:** Naranjo scale score of fulminant multiorgan failure caused by tislelizumab.

Are there previous conclusive reports on this reaction?	Yes, +1
Did the adverse event appear after the suspected drugs was given?	Yes, +2
Did the adverse reaction improve when the drug was discontinued or when a specific antagonist was given?	Not known or not done, 0
Did the adverse reaction appear when the drugs was readministered?	Not known or not done, 0
Are there alternative caused the reaction?	Yes, +2
Did the reaction reappear when a placebo was given?	Not known or not done, 0
Was the drug detected in any body fluid in toxic concerntrations?	Not known or not done, 0
Was the reaction more serve when the dose was increased, or less serve when the dose was decreased?	No, 0
Did the patient have a similar reaction to the same or similar drugs in any previous explorsure?	No, 0
Was the adverse event confirmed by any objective evidence?	Yes, +1
Total	6

The patient had no underlying conditions for cirrhosis or acute liver failure. Acute progression of cholangiocarcinoma itself was ruled out as highly unlikely and because the patient’s imaging studies on admission did not show progression.

Although sepsis can also present with high fever, markedly elevated PCT, and subsequent positive pathogen serology, the timing of symptoms is too strongly correlated with Immunotherapy (within hours), and sepsis rarely causes injury in the pattern of AST >2000 U/L and myoglobin >1000 ng/mL, the condition continues to worsen after high-dose steroid pulse. It is also not completely consistent with typical sepsis. We do not dispute the diagnosis of sepsis, but it is not the underlying cause of this fatal event. We believe that sepsis is more like an integral part of the disease process, with the underlying driver being a lethal immune storm triggered by immunotherapy. Influenza virus infection may act as an immune adjuvant, exacerbating this process.

Previous reports have associated tislelizumab with adrenal insufficiency, psoriasis, liver injury, and diabetic ketoacidosis, as well as severe thyrotoxicosis, cytokine release syndrome, toxic epidermal necrolysis-like skin reactions, steroid-refractory immune checkpoint inhibitor-related pneumonitis, acetylcholine receptor-binding antibody-associated myasthenia gravis, myocarditis, rhabdomyolysis, and pancytopenia. There have been no reported fatalities associated with tislelizumab (**Table 3**).

**Table 3:** Tislelizumab-related adverse events in available case reports.

Malignancies	Grender	Age	Diagnosis	Reference
Lung cancer	M	72	cytokine release syndrome	(10)
urothelial carcinoma	M	58	Adrenal crisis	(11)
thymic carcinoma	M	27	ureteritis/cystitis	(12)
dual organs dysfunction	M	74	acute kidney injury (grade 3) and acute liver injury (grade 4)	(13)
bladder cancer	M	67	adrenal hypofunction and Psoriasisby induced by tislelizumab	(14)
Liver cancer	M	49	Severe thyrotoxicosis	(15)
Bladder Cancer and Prostate Cancer	M	72	Hypophysitis	(16)
Chronic nonspecific cheilitis	F	36	Chronic nonspecific cheilitis	(17)
lung adenocarcinoma	M	66	Life-threatening pancytopenia	(18)
metastatic lung cancer	F	75	Lichen planus pemphigoides	(19)
metastatic Lung Squamous cell Carcinoma (LUSC)	M	75	Toxic Epidermal Necrolysis and Agranulocytosis	(20)
Esophageal cancer	M	63	Exfoliative esophagitis	(21)
pulmonary sarcomatoid carcinoma	M	59	Fatal hemoptysis	(22)
thymic epithelial tumors	M	42 and 52	Severe cardiotoxicity	(23)
Non-Small Cell Lung Cancer	M	71	multiple-organs irAEs (lung,muscle,myocardium,liver,and pituitary)	(24)
Lung cancer	M	74	THSD7A-Positive Membranous Nephropathy	(25)
colon cancer	M	65	Acetylcholine receptor binding antibody-associated myasthenia gravis,myocarditis,and rhabdomyolysis	(26)

The pathophysiological mechanism of this fatal event involves the synergy between ICI therapy and viral infection: influenza virus can upregulate PD-L1 expression (27), which can regulate the host immune response during infection by this mechanism (28), as an adaptive immune resistance mechanism to inhibit T cell responses. ICI treatment,by blocking PD-1/PD-L1,a key immunosuppressive checkpoint,reversed depletion of cytotoxic T lymphocytes (CTLS) and promoted their proliferation and activation (29). During acute infection,upregulation of PD-1 on CD8^+^ T cells serves as a host feedback inhibitory mechanism to prevent excessive immunopathology. Meanwhile,hepatic sinusoidal endothelial cells constitutively express high levels of PD-L1,which promotes immune tolerance and protects hepatic tissue from immune damage through activation of the PD-1/PD-L1 checkpoint pathway (30). ICI therapy abrogates this protective mechanism,leading to severe immune-related adverse events (irAEs) through disinhibition of regulatory signaling and enhanced immune activation.

In this case, immune-mediated hepatitis was the primary clinical manifestation. Within four days of treatment, he developed fulminant hepatic injury concurrent with acute kidney injury(KDIGO stage 3). The patient also developed bone marrow suppression, myositis, and renal impairment, culminating in fatal multisystem organ failure. The particular nature and high severity of these irAEs have not been previously reported in clinical trials of tislelizumab.

We speculate that influenza virus infection may have served as a critical trigger. During viral infection, upregulated PD-1 expression on CD8^+^T cells functions to suppress excessive immune activation. The excessive immune response is caused by the suppression of PD-1-mediated immune tolerance by ICI treatment, which eventually leads to liver failure. Although liver replacement therapy might have been the only potential rescue intervention, it was not feasible in this case.

## Limitations

In this case report, influenza virus PCR testing on respiratory samples was not performed. As this is a retrospective analysis, clinical management during the acute presentation was primarily focused on stabilizing the patient’s rapidly deteriorating condition (e.g. fulminant hepatic failure). Thus, the patient had severe thrombocytopenia and coagulopathy, and a liver biopsy was not performed because it was considered to be a very high risk of bleeding. Furthermore, a postmortem examination (autopsy) was not performed, as it was declined by the family. Serologic antibody testing and routine biochemical and inflammatory markers were prioritized to facilitate rapid diagnosis and guide urgent treatment decisions. When the patient developed hyperpyrexia and respiratory symptoms,clinicians strongly suspected viral infection based on the clinical presentation (e.g. high fever, catarrhal symptoms) and epidemiological context. Although PCR confirmation was unavailable,empirical treatment with the antiviral agent baloxavir marboxil was promptly initiated in accordance with the principle of “clinical diagnosis first”. This approach aligns with standards of care for critically ill patients, where in treatment should not be delayed pending definitive laboratory results. But in future research, it is also advised that respiratory samples undergo influenza PCR testing and liver biopsy to elucidate the role of influenza virus infections in the patients’ prognosis.

## Conclusion

This case report describes a 72-year-old male with postoperative cholangiocarcinoma who developed fulminant multiorgan immune-related adverse events (irAEs) shortly after initiating treatment with tislelizumab combined with anlotinib. Although immune-mediated hepatitis associated with tislelizumab has been reported, it is rarely fatal. In this patient, concomitant influenza virus infection may have contributed to a fulminant course through immune hyperactivation. Clinicians should be aware of this potentially life-threatening toxicity. Further research is needed to determine how to use ICI in patients with concurrent viral infections and to minimize risks.

## Data Availability

The raw data supporting the conclusions of this article will be made available by the authors, without undue reservation.
